# US Hospital Capacity Managers’ Experiences and Concerns Regarding Preparedness for Seasonal Influenza and Influenza-like Illness

**DOI:** 10.1001/jamanetworkopen.2021.2382

**Published:** 2021-03-19

**Authors:** Gavin H. Harris, Kimberly J. Rak, Jeremy M. Kahn, Derek C. Angus, Olivia R. Mancing, Julia Driessen, David J. Wallace

**Affiliations:** 1 Division of Pulmonary, Allergy, and Critical Care Medicine, Department of Medicine, Emory University School of Medicine, Atlanta, Georgia; 2 Division of Infectious Diseases, Department of Medicine, Emory University School of Medicine, Atlanta, Georgia; 3 Department of Critical Care Medicine, University of Pittsburgh School of Medicine, Pittsburgh, Pennsylvania; 4 Department of Health Policy and Management, University of Pittsburgh Graduate School of Public Health, Pittsburgh, Pennsylvania; 5 Department of Emergency Medicine, University of Pittsburgh School of Medicine, Pittsburgh, Pennsylvania

## Abstract

**Importance:**

The 2017-2018 influenza season in the US was marked by a high severity of illness, wide geographic spread, and prolonged duration compared with recent previous seasons, resulting in increased strain throughout acute care hospital systems.

**Objective:**

To characterize self-reported experiences and views of hospital capacity managers regarding the 2017-2018 influenza season in the US.

**Design, Setting, and Participants:**

In this qualitative study, semistructured telephone interviews were conducted between April 2018 and January 2019 with a random sample of capacity management administrators responsible for throughput and hospital capacity at short-term, acute care hospitals throughout the US.

**Main Outcomes and Measures:**

Each participant’s self-reported experiences and views regarding high patient volumes during the 2017-2018 influenza season, lessons learned, and the extent of hospitals’ preparedness planning for future pandemic events. Interviews were recorded and transcribed and then analyzed using thematic content analysis. Outcomes included themes and subthemes.

**Results:**

A total of 53 key hospital capacity personnel at 53 hospitals throughout the US were interviewed; 39 (73.6%) were women, 48 (90.6%) had a nursing background, and 29 (54.7%) had been in the occupational role for more than 4 years. Participants’ experiences were categorized into several domains: (1) perception of strain, (2) effects of influenza and influenza-like illness on staff and patient care, (3) immediate staffing and capacity responses to influenza and influenza-like illness, and (4) future staffing and capacity preparedness for influenza and influenza-like illness. Participants reported experiencing perceived strain associated with concerns about preparedness for seasonal influenza and influenza-like illness as well as concerns about staffing, patient care, and capacity, but future pandemic planning within hospitals was not reported as being a high priority.

**Conclusions and Relevance:**

The findings of this qualitative study suggest that during the 2017-2018 influenza season, there were systemic vulnerabilities as well as a lack of hospital preparedness planning for future pandemics at US hospitals. These issues should be addressed given the current coronavirus disease 2019 pandemic.

## Key Points

**Question:**

What were US hospitals’ experiences and the views of hospital capacity managers regarding response and preparedness activities during the 2017-2018 influenza epidemic?

**Findings:**

In this qualitative study using semistructured telephone interviews with 53 key hospital capacity personnel at 53 hospitals throughout the US, perceived strain on hospital resources was almost universally reported. Participants described a range of hospital responses to seasonal influenza but indicated that future pandemic planning was not a high priority.

**Meaning:**

The findings suggest that, during the 2017-2018 influenza epidemic, there were vulnerabilities in the US health care system, including a lack of planning for future pandemic events, which may have implications for public health planning given the ongoing coronavirus disease 2019 pandemic.

## Teaser

This qualitative study uses interviews with capacity management personnel in US acute care hospitals during the 2017-2018 influenza season to examine views regarding hospitals’ responses to high patient volumes and the level of hospital preparedness for future pandemic events.

## Introduction

The 2017-2018 influenza season in the US was marked by a high severity of illness, wide geographic spread, and prolonged duration.^1^ Rates of hospitalization in all age groups were the highest observed since seasonal influenza surveillance was instituted in 2005. This was associated with substantial increases in patient volumes in hospitals and hospital systems, which led to disruptions in flow, patient care, and staffing.^2^ The US Centers for Disease Control and Prevention estimated that influenza alone was associated with more than 27.7 million medical visits, 959 000 hospitalizations, and 79 400 deaths in 2018.^3^

The Centers for Disease Control and Prevention and the Department of Health and Human Services released an updated National Influenza Pandemic Preparedness Plan in 2017 that identified hospital operations, patient care, and staffing as essential domains that facilities must address to prepare for future pandemic events.^4^ The document provided guidance on health care system preparedness and response activities, emphasizing the importance of implementing surge strategies for conditions such as influenza or other severe community-based infectious diseases. The recommendations, along with the Crisis Standards of Care produced by the National Academy of Medicine, provide hospitals with a framework for developing, prioritizing, and implementing disaster response plans.^5^ Professional societies have also provided hospital recommendations for preparedness related to influenza or mass disaster events.^6,7,8,9,10,11^ However, to date, the extent of implementation of these recommendations into contingency planning for short-term acute care hospitals is not known.^12,13^

The objective of this study was to examine self-reported experiences and views of hospital capacity managers regarding sustained high patient volumes during the 2017-2018 influenza season, to identify lessons learned, and to assess the extent of preparedness planning for future pandemic events. The study results have implications for public health planning given the ongoing coronavirus disease 2019 (COVID-19) pandemic and may contribute to understanding how hospitals and hospital systems have responded and are continuing to respond to the pandemic.

## Methods

### Study Design

In this qualitative study, we conducted semistructured telephone interviews with capacity management personnel in short-term acute care hospitals in the US. Study participants provided oral informed consent to be interviewed, to have interviews recorded and transcribed, and to have direct quotes published from the interviews. Participants were informed that personal or institution-identifying elements from the interview would be redacted from interview transcripts and would not be published. The University of Pittsburgh institutional review board determined the study to be exempt from the need for institutional review board approval because it met all criteria for exemption per the 45 CFR 46.104[d]. This study followed the Consolidated Criteria for Reporting Qualitative Research (COREQ) reporting guideline.^14^

### Participants

We identified a random sample of short-term acute care hospitals with intensive care unit (ICU) beds using the Centers for Medicare & Medicaid Services’ Healthcare Cost Report Information System (HCRIS), a publicly available annual report generated by hospitals that submit claims to the Centers for Medicare & Medicaid Services. All 3111 short-term acute care hospitals that reported having ICU beds and a telephone contact number in HCRIS were eligible.

Pursuant to a separate research question focused on factors associated with changes in the numbers of ICU beds in hospitals or hospital systems, we used stratified random sampling by 2 hospital characteristics—annual ICU occupancy and 5-year changes in the numbers of ICU beds—to produce 6 sample frame categories. We used the main telephone number listed in the HCRIS to identify an administrator responsible for capacity management or throughput, and in all cases, this number reached a hospital operator. Purposeful sampling was used to find an appropriate participant for the interview who self-identified as being able to answer questions related to hospital capacity.^15^ After identifying a candidate participant, we scheduled a time for the study interview with 1 of 4 members of the study team (K.J.R., O.R.M., J.D., or D.J.W.). One interviewer was an experienced medical anthropologist (K.J.R.), and the remaining interviewers (O.R.M., J.D., and D.J.W.) received qualitative-interview training before conducting study interviews. In several sessions, the staff medical anthropologist reviewed interviewing standards of practice with the other interviewers. In addition, the staff medical anthropologist joined the first 3 interviews conducted by each of the other interviewers and provided feedback during and after these sessions. Finally, in reviews of transcripts, all senior members of the team identified sections of each interview that could be improved. Participation was limited to 1 interview per hospital, and $50 compensation was offered for participation. Participants were noted to have a variety of backgrounds, from nursing to hospital administration.

### Data Collection

Four of the authors (K.J.R., O.R.M., J.D., and D.J.W.) conducted telephone interviews with participants using an interview guide between April 2018 and January 2019. The interview guide was based on a literature review of hospital-strain research and sequential feedback from 8 rounds of pilot testing with capacity managers at 8 hospitals. Following each pilot interview, questions were reviewed with the participant, soliciting feedback on clarity and flow.^16,17,18^ The following topical areas were covered in the interview guide: participants’ role in the hospital and how it related to bed capacity; resources available for patient flow; definition of high census; operational procedures related to high census; areas of patient-flow bottlenecks; changes (or lack thereof) in number of ICU beds; strain in the ICU; perceptions of the response to and impact of the 2017-2018 flu season; the effect of strain on quality of care and staff well-being; and future planning. Study interviews were recorded, transcribed, and analyzed using a constant comparative approach. This approach allowed for the refinement of themes as they emerged during successive interviews by comparing existing data with new data. The study team met on a weekly basis to review interviews and modify the interview guide as cohesive themes became apparent. Member checking was performed during interviews through summarization of participant responses as a narrative accuracy check to minimize incorrect interpretation of data.^19,20,21^ A sample size was not set at the outset of the study, although approximately 30 to 40 interviews were anticipated to be needed to reach thematic saturation based on previous experience.^22,23,24^ Transcripts were not returned to participants for review. Thematic saturation was achieved after 53 interviews. Interview duration ranged from 45 minutes to 1 hour.

### Analysis

Coding and data analysis were done between March and August 2019 using NVivo qualitative data analysis software, version 12 (QSR International). Two authors (K.J.R. and D.J.W.) read transcripts, coded concepts, organized concept relationships into themes and subthemes, and developed a codebook through discussion and consensus with the study team. Two coders (K.J.R. and D.J.W.) met weekly to review interrater reliability, which was assessed through an NVivo-generated report of code-specific percentage agreements and κ statistics. Any codes with less than 90% agreement were reviewed and resolved by consensus. A total agreement percentage greater than 90% (with a corresponding κ value of 0.63) was achieved after co-coding 6 interviews (11% of the 53 total interviews). Once we obtained acceptable interrater reliability, interviews were coded by 1 of the 2 coders (K.J.R. or D.J.W.) with the exception of 1 co-coded interview every 10 interviews to check continued coding agreement. Another round of review and consensus occurred for any codes that had less than 90% agreement.

## Results

From the 101 hospitals contacted, 53 capacity managers at 53 hospitals were identified and agreed to participate; all 53 were interviewed, and 53 audio transcripts were included in the final analysis, for a response rate of 52.5%. Among the 53 participants, 47 (88.7%) identified as non-Hispanic White, 39 (73.6%) were women, and 48 (90.6%) reported an occupational background in nursing. Most of the participants (29 [54.7%]) reported being in their occupational role for more than 4 years, and 29 (54.7%) reported working in health care for more than 20 years (Table 1). Participant hospitals were located in 25 states and in all geographic regions of the US (Figure). The bed capacity of the participant hospitals ranged from less than 250 beds (13 hospitals [24.5%]) to 500 beds or more (19 hospitals [35.8%]), with most hospitals (31 [58.5%]) reporting more than 50 total ICU beds (Table 2).

**Table 1.** Characteristics of Interviewed Participants

Characteristic	Participants, No. (%) (N = 53)
Women	39 (73.6)
Men	14 (26.4)
Race/ethnicity	
Hispanic	0
Non-Hispanic White	47 (88.7)
African American	3 (5.7)
Asian	3 (5.7)
Occupational background	
Nursing	48 (90.6)
Other	5 (9.4)
Professional title	
Logistics personnel	15 (28.3)
Administrator	15 (28.3)
Nursing supervisor	7 (13.2)
Administrative supervisor	7 (13.2)
Bed capacity management personnel	5 (9.4)
Other	4 (7.5)
Duration of occupation in current position, y	
<2	8 (15.9)
2-4	16 (30.2)
>4	29 (54.7)
Duration of occupation in health care, y	
<10	2 (3.8)
10-20	22 (41.5)
>20	29 (54.7)

**Figure.**
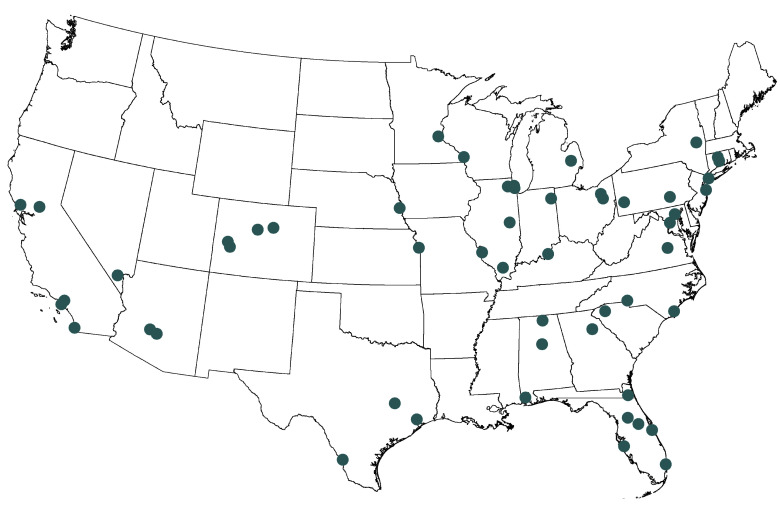
Participant Hospital Locations in the US Circles show locations of participating hospitals.

**Table 2.** Characteristics of Participants’ Hospitals

Characteristic	Participants, No. (%) (N = 53)
Hospital beds	
<250	13 (24.5)
250-499	21 (39.6)
>499	19 (35.8)
ICU beds	
≤20	11 (20.8)
21-50	11 (20.8)
>50	31 (58.5)
ICU occupancy	
Higher occupancy^a^	27 (50.9)
Lower occupancy^b^	26 (49.1)
Change in number of ICU beds in past 5 y	
Increasing ICU bed supply	11 (20.8)
Minimal change in ICU bed supply	23 (43.4)
Decreasing ICU bed supply	19 (35.8)
Teaching status	
Nonteaching	19 (35.8)
Minor teaching	16 (30.2)
Major teaching	18 (34.0)
Financial status	
For-profit	10 (18.9)
Nonprofit	37 (69.8)
Government	6 (11.3)
Critical access status	0
Urban location	48 (90.6)
Geographic region	
Northeast	7 (13.2)
Midwest	14 (26.4)
West	12 (22.6)
South	20 (37.7)

Abbreviation: ICU, intensive care unit.

^a^ Mean annual ICU occupancy greater than 80%.

^b^ Mean annual ICU occupancy less than or equal to 80%.

We identified 4 overall themes from participant interviews: (1) perceptions of strain, (2) effects of influenza and influenza-like illness on staff and patient care, (3) immediate responses to influenza and influenza-like illness, and (4) future preparedness for influenza and influenza-like illness. Descriptions of each key theme and subtheme are provided in the following sections. Representative quotations to support each subtheme are provided in the text and in Table 3.

**Table 3.** Themes, Subthemes, and Illustrative Quotations From Interviews

Theme	Illustrative quotation^a^
Perceptions of strain	“This is the highest influx of flu that was admitted that I’ve seen in probably 10, 12 years. We were really hit hard, and that again put more strains on the resources because they had to be isolated.” “Flu created strain on the entire hospital just because of the sheer volume of patients.” “The NEDOC score and our capacity probably hit the highest that they’ve ever seen in the past 20 years.”
Impacts of influenza	
Staff impact	“The increased patient volumes ended up becoming a physician capacity issue. Our hospitalist group became overwhelmed with the influx of patients.” “We also see the burnout because then what’s left behind is the nurses who haven’t gotten sick but are trying to pick up for those who are not here.” “When you’re going through that many more, it also affects your EVS staff…and then you don’t have rooms cleaned as fast. And your kitchen staff.”
Patient care impact	“We ended up boarding more patients in our emergency department than we ever had in the past and we had to support staffing to do that…But what would happen if we got to a point where we were so overrun, we would have to slow down our ORs?…So, you would stop doing inpatient surgery on patients that would need beds…like you would have to make very, very hard decisions.” “We were as high as having like 48 people isolated at a time. And we don’t have 48 private rooms. So that means we had to take semiprivates and make them private, which further impacted our ability to do flow.” “It…put a big strain on our isolation supplies and our isolation rooms.”
Immediate responses	
Staffing responses	“We ended up needing...mandatory overtime. We had staff sign up. We asked everyone to work 1 extra shift per pay period. So that kind of took its toll on staff as well. It wasn’t really a big satisfier, but we needed to do that in order to make sure all the patients were cared for safely.” “We do have a 10-bed overflow unit, and that was open all fall, all winter long to help with increased patient load. We did utilize some temporary staff agency; contractual staff to help meet the need of our increased patient census.”
Capacity responses	“We do have surge plans in place. Do people follow them? No.” “We have a surge plan that we use that changes…during the day. We implemented that a number of times during that period to get discharges out sooner.” “We had our hospital system’s ambulance service...actually taking patients out of the ED...off the floors as well that were ready for discharge.”
Future preparedness	
Staff	“We’re already hiring travelers and getting everything set up. Like I said, we’re setting up the units. We want to be prepared to deliver that same level of service we expect on the floors. And really you need people to be able to do that so we’re hiring enough temporary help to manage us getting sick and the amount of people that come through our doors that are sick.” “In our lower times we really try and proactively cross train people to a higher level of care and give them the experience that they need so that when the need does arise, we’re prepared for it.”
Hospital capacity	“This last one challenged us where we’ve changed our policies, and that’s where that new capacity management plan came from.” “We haven’t been able to do too much else with bed planning because we pretty much just tapped out everything you could think of as far as areas to go. So we’ll continue to keep brainstorming, but we’ve already tapped into every little place you could think of.” “We have just put together a high census committee and we are working on several action plans...one of those being what we consider overwhelming at the hospital. Just diversion is pretty much like a 4-letter word, and nobody wants to use it. We’re thinking of better ways to go about communicating with EMS and other facilities...So we are actually putting together an action plan now.”

Abbreviations: ED, emergency department; EMS, emergency medical services; EVS, environmental services; NEDOC, National Emergency Department Overcrowding Scale; OR, operating room.

^a^ Quotations are presented verbatim.

### Perceptions of Strain

All participants reported experiencing hospital strain during the 2017-2018 influenza season. Strain was generally described as the experienced consequences of high occupancy when demand for resources was, either in fact or in perception, greater than the resources available. Most participants described strain as existing on a continuum, with increasing impacts at greater levels of strain intensity. Many participants reported using either locally developed measures or the National Emergency Department Overcrowding Scale score for quantifying strain.

### Perceptions of Impacts of Influenza and Influenza-like Illness

Substantially increased numbers of patients seeking care for influenza and influenza-like illness had a variety of effects on health care delivery throughout the seasonal surge. Two significant subthemes were reported by participants: effect on hospital staff and effect on patient care.

#### Hospital Staff

Participants reported that influenza and influenza-like illness had several consequences for hospital staff. First, increased patient volumes were associated with strain and fatigue among frontline health care practitioners through the provision of increased medical care. Participants reported needing to adjust ratios of practitioners and nurses to patients in response to patient volume. Second, capacity management strategies to mitigate the effects of increased patient volume created additional burdens on staff. Participants reported that additional administrative efforts to address capacity resulted in recurrent surge meetings and bed flow adjustments, creating “very taxing pressure, constantly thinking of solutions.” Third, influenza directly affected staff who became sick. Fourth, staffing shortages owing to illness or provision of child care amplified strain on remaining staff members. A capacity management administrator commented that burnout became a real concern. In addition, some participants reported an effect of strain on nonclinical hospital positions.

#### Patient Care

Many participants perceived negative outcomes for patient care. Influenza strained infrastructure through increased need for respiratory isolation and increased burden on physicians and nurses. An increase in hospital diversion and boarding for emergency departments was almost universally reported. A decrease in elective operations was reported by some participants. At critical levels of strain, some participants reported an effect of strain on health care quality as well as concerns related to the transfer of patients out of the ICU sooner than when the hospital was not under strain.

Many participants commented on impacts related to supplies and equipment. One capacity manager reported, “It…put a big strain on our isolation supplies and our isolation rooms.” Another said, “It affects dietary trying to deliver trays or figure out where, you know...how do you put in diet orders for a unit that you didn’t even have built?”

### Immediate Responses to Influenza and Influenza-like Illness

Respondents reported a variety of immediate responses to influenza and influenza-like illness. The 2 dominant subthemes were immediate staff responses and immediate hospital capacity responses.

#### Staff Responses

Respondents reported a range of mechanisms to increase or maintain clinical staffing during the influenza season. To address staffing shortfalls, hospitals engaged agency services, travel-nursing resources, and temporary nursing services and reallocated nurses within hospital systems. Some respondents reported that shift allocation policies were adapted to provide additional staff during the influenza season. One manager also reported that new mandatory overtime policies were necessary, along with new influenza vaccination policies for staff.

#### Hospital Capacity Responses

Most respondents reported that new policies or protocols were implemented to improve throughput. These policies and protocols involved the use of existing clinical space in new ways and the transformation of some previously nonclinical areas in the hospital into spaces for clinical care. Many respondents reported that new clinical areas were opened for cohorts of suspected and confirmed influenza cases. Most respondents reported that regular administrative meetings were held to evaluate capacity and throughput during the influenza season. Some respondents reported that lower priority meetings were cancelled during influenza and that practitioners “[were] not going to meetings, and really focusing on clinical work to get the patients moved.”

Participants did not universally report implementation of hospital plans or protocols based on perceptions or measures of overcrowding. In response to actions taken at the highest level of overcrowding measure, 1 nurse manager commented that while surge plans were in place, buy-in proved difficult, creating barriers to implementation. Owing to shortcomings, some hospitals had to resort to creative strategies to mitigate the impacts of the surge. One participant reported that “we had our hospital system’s ambulance service…actually taking patients out of the ED [emergency department] [and] off the floors as well that were ready for discharge.”

### Future Preparedness

Few participants reported future hospital-based preparedness activities, citing only actions that were taken during the 2017-2018 influenza season. When reported, however, future preparedness was mainly confined to only 2 subthemes: future staffing preparedness and future hospital operation preparedness.

#### Staff Preparedness

Some participants reported anticipating changes that would be intended to maintain a sufficient clinical workforce during the upcoming influenza season. Alterations to sick-call policies and to the use of travel-nursing resources were reported. Participants also reported cross-training nurses in preparation for future events. One capacity manager commented, “In our lower times we really try and proactively cross train people to a higher level of care and give them the experience that they need so that when the need does arise, we’re prepared for it.”

#### Hospital Capacity Preparedness

Some participants reported that new protocols were planned for subsequent events. After the 2017-2018 influenza season, several respondents reported new “census committees,” action plans, and better communication with prehospital emergency medical services. Few participants reported the use of surge scenarios to evaluate the capacity of the hospital to handle a large increase in patient numbers. One capacity manager reported, “Some of the scenarios that we came up with was where can we isolate patients. This has been there from H1N1. We had to develop a lot of these things and we just keep them in our back pocket for when we need them.” Many participants reported plans to expand the number of total beds or ICU beds in their hospital, although none specifically identified influenza preparedness as the motivation for the planned construction.

## Discussion

The 2017-2018 influenza season caused strain on the US health care system through a prolonged surge of patients and overwhelmed hospital capacities in many parts of the country. In this qualitative study, we explored how sustained strain impacted the experiences and views of hospital capacity management personnel, how they responded to such challenges in the short term, and how lessons learned translated into preparedness for future pandemics. We found that hospitals experienced substantial perceived strain as an outcome of the 2017-2018 influenza season, which was associated with widespread self-reported consequences on the “4 S’s”: staff, stuff, space, and systems.^25,26,27,28^ The findings are of particular interest given the widespread challenges experienced by hospitals in response to the COVID-19 pandemic.

The results of this study suggest that although strain from influenza and influenza-like illness was perceived to different extents and in different ways among hospital capacity managers, the 2017-2018 influenza season was critically taxing for most and for a longer duration than prior influenza seasons. Many hospitals experienced staffing crises and had substantial challenges in maintaining the nursing workforce, problems exposed by the current shortage and maldistribution of nurses in the US (although this is not uniquely a US problem).^29,30,31^ Although many participants described an effect of sustained strain from influenza and influenza-like illness on staff well-being, safety concerns from infection were not reported as a substantial cause of missed work. It is possible that the 2017-2018 outbreak registered as a less significant personal safety threat for health care workers compared with the 2009 influenza A (H1N1) pandemic, but hospitals should anticipate, and are already seeing, safety concerns among frontline staff in response to COVID-19.^32^ The COVID-19 pandemic has been exerting a heavy toll on both health care workers and the health system.^33^ Of note, the perceptions of strain on staffing, patient care, and capacity that we have seen during the COVID-19 pandemic were already present with prior epidemics.

In fall 2013, the US Department of Health and Human Services Office of the Assistant Secretary for Preparedness and Response (ASPR) produced the Interim Healthcare Coalition Checklist for Pandemic Planning report,^34^ which identified capabilities in 8 categories that hospitals should address when planning for crises: health care system preparedness, health care system recovery, emergency operations coordination, fatality management, information sharing, medical surge, responder safety and health, and volunteer management. Although some domains were described by all participants in this study, no participant commented on all domains, nor did participants specifically report the use of the ASPR checklist for capacity management or pandemic planning. In particular, fatality management, responder safety and health, and volunteer management were not described by respondents. The ASPR has also developed 2 tools to evaluate hospital capacity: the Hospital Surge Evaluation Tool^35^ and the Health Care Coalition Surge Test.^25^ The Hospital Surge Evaluation Tool is a detailed exercise designed to help identify gaps in a hospital’s preparedness and assess its ability to respond to a mass casualty incident.^35^ The Health Care Coalition Surge Test is a low- or no-notice exercise designed to identify gaps in hospital surge planning.^25^ Responses from participants in the current study indicate that although the current US health care system had some resilience for increasing patient volumes, there were few examples of hospitals pursuing comprehensive capacity-expanding plans based on the ASPR checklist, using the ASPR Hospital Surge Evaluation Tool or Health Care Coalition Surge Test, or explicitly describing future preparedness activities in response to lessons learned from the 2017-2018 season.^26^ Outside the Society for Critical Care Medicine, there has been a paucity of disaster response guidance, which may account for the lack of weight that pandemic planning has had in hospital contingency planning. This study highlights the urgent need for emphasis on future events.

### Strengths and Limitations

This study has strengths. The qualitative approach of this study was strengthened by the use of member checking, constant comparative analysis, and thematic saturation, which produced detailed descriptions from the perspective of key hospital informants. The study also has limitations. It was designed and implemented as a qualitative analysis to examine the perceptions and experiences of hospital capacity managers, but it did not examine the actual effects, responses, and preparedness activities among hospitals. In addition, the effectiveness of reported preparedness interventions was not studied, and hospital quality was not included in the analysis.

## Conclusions

This qualitative study generated insights into capacity management during the 2017-2018 influenza season at short-term acute care hospitals in the US. Although hospitals implemented a range of immediate responses, they generally did not report future planning specific to a pandemic event. This study’s results suggest that the threat of a future pandemic was underestimated despite the presence of warning signs during the 2017-2018 influenza season and that the subsequent consequences for staffing and patient care were frequently underappreciated. In the past year, these vulnerabilities have come to the forefront, and it is essential that any hospital or hospital system take into account all aspects of capacity not only in combating the current pandemic but also in planning for future events.
